# *Propionibacterium acnes*-Derived Circulating Immune Complexes in Sarcoidosis Patients

**DOI:** 10.3390/microorganisms9112194

**Published:** 2021-10-21

**Authors:** Keisuke Uchida, Asuka Furukawa, Akiko Yoneyama, Haruhiko Furusawa, Daisuke Kobayashi, Takashi Ito, Kurara Yamamoto, Masaki Sekine, Keiko Miura, Takumi Akashi, Yoshinobu Eishi, Kenichi Ohashi

**Affiliations:** 1 Division of Surgical Pathology, Tokyo Medical and Dental University Hospital, Tokyo 113-8510, Japan; uchida.path@tmd.ac.jp (K.U.); mspath@tmd.ac.jp (M.S.); k.miura.pth1@tmd.ac.jp (K.M.); akashi.path@tmd.ac.jp (T.A.); 2 Department of Human Pathology, Graduate School and Faculty of Medicine, Tokyo Medical and Dental University, Tokyo 113-8510, Japan; a.tajima.pth1@tmd.ac.jp (A.F.); d-koba.pth1@tmd.ac.jp (D.K.); t.ito.pth1@tmd.ac.jp (T.I.); kakipth1@tmd.ac.jp (K.Y.); kohashi.pth1@tmd.ac.jp (K.O.); 3 Division of Nutrition Services, Tokyo Medical and Dental University Hospital, Tokyo 113-8510, Japan; akiko.y.nutr@tmd.ac.jp; 4 Department of Integrated Pulmonology, Graduate School and Faculty of Medicine, Tokyo Medical and Dental University Hospital, Tokyo 113-8510, Japan; hfurusawa.pulm@tmd.ac.jp

**Keywords:** sarcoidosis, *Propionibacterium acnes*, *Cutibacterium acnes*, lipoteichoic acid, infectious antibody, immune complex, antigen retrieval, sandwich ELISA

## Abstract

*Propionibacterium acnes* is a potential etiologic agent of sarcoidosis and a dysregulated immune response to the commensal bacterium is suspected to cause granuloma formation. *P. acnes*-derived insoluble immune complexes were recently demonstrated in sinus macrophages of sarcoidosis lymph nodes, suggesting local proliferation of the bacterium in affected organs. In the present study, we developed a method for detecting *P. acnes*-derived immune complexes in human blood by measuring the concentration of *P. acnes*-specific lipoteichoic acid (PLTA) detectable after an antigen retrieval pretreatment of plasma samples. Before pretreatment, anti-PLTA antibody was detected and PLTA could not be detected, in all plasma samples from 51 sarcoidosis patients and 35 healthy volunteers. After pretreatment, however, a significant level of PLTA (>105 ng/mL) was detected in 33 (65%) sarcoidosis patients and 5 (14%) control subjects, with 86% specificity and 65% sensitivity for sarcoidosis. In both groups, plasma anti-PLTA antibody titers did not differ between samples with and without detection of PLTA. PLTA levels were abnormally increased (>202 ng/mL) in 21 (41%) sarcoidosis patients. These findings suggest that *P. acnes*-derived circulating immune complexes present in human blood are abnormally increased in many sarcoidosis patients, presumably due to local proliferation of the bacterium in the affected organs.

## 1. Introduction

Sarcoidosis is a multisystemic granulomatous disease of unknown cause [1]. The histologic hallmark of sarcoidosis is non-caseating epithelioid cell granulomas, which can affect virtually any organ in the body, including the lungs, lymph nodes, skin, eyes, or a combination of these sites. Granulomatous inflammation in sarcoidosis is thought to be a dysregulated antigenic response to unknown environmental exposure in a genetically susceptible person [2]. The environmental factors described as potential causes of sarcoidosis include a large list of antigens that may derive from infectious agents (e.g., Mycobacteria, Propionibacteria, fungi, and viruses) or inorganic compounds (e.g., zirconium, aluminum, and occupational exposures) [3,4].

Among the potential infectious agents, *Propionibacterium acnes* (currently referred to as *Cutibacterium acnes*) [5] is the only microorganism that has been isolated from sarcoidosis lesions to date [6,7]. Large amounts of *P. acnes* DNA are detected by quantitative polymerase chain reaction in sarcoidosis tissues, suggesting that this bacterium proliferates at the site of disease activity [8,9]. In situ hybridization and immunohistochemical methods demonstrate the bacterial DNA or antigens in sarcoidosis granulomas [10,11], suggesting a histopathologic link of the commensal bacterium to the cause of granuloma formation: *P. acnes* has been detected in granulomas of some sarcoidosis patients, but not in any non-sarcoidosis granulomas, including tuberculosis and sarcoid reaction granulomas [11,12]. In some sarcoidosis patients, immune responses to certain *P. acnes* antigens are increased [13,14,15,16] and an imbalance of Th1/Th17 immune responses to the commensal bacterium is suggested [14]. Thus, *P. acnes* may cause granuloma formation in some predisposed individuals by a dysregulated immune response against intracellular proliferation of the bacterium triggered by certain host-related or drug-induced conditions [17,18,19].

Circulating immune complexes in sarcoidosis patients were reported by many authors [20,21,22,23,24,25] from 1974 to 1980, and insoluble immune complexes in sarcoidosis lymph nodes were first reported by Eishi et al. [26] in 1988. While the etiology and role of these immune complexes in sarcoidosis has remained unknown for a long time, Suzuki et al. [27] recently demonstrated many *P. acnes*-derived insoluble immune complexes in sinus macrophages of sarcoidosis lymph nodes by immunohistochemistry with a monoclonal antibody that reacts with *P. acnes*-specific lipoteichoic acid (PLTA) localizing on the bacterial cell membrane. PLTA is a major *P. acnes*-specific antigen; the monoclonal anti-PLTA antibody obtained by immunizing mice with *P. acnes* does not cross-react with other propionibacterial species such as *P. granulosum* or other gram-positive bacteria such as mycobacteria [11].

Detection of *P. acnes* bound with immunoglobulins by immunohistochemistry requires an antigen retrieval pretreatment (brief trypsin digestion of tissue sections after microwaving) to separate immunoglobulins from the immune complexes and allow the primary antibody to bind with the exposed PLTA antigen, which is resistant to trypsin digestion [27]. Thus, in their experiments, the immunohistochemical detection of the PLTA antigen retrieved by the pretreatment was abolished by incubating the tissue sections with human plasma before the primary antibody reaction. These observations suggest that plasma anti-PLTA antibodies compete with the primary antibody used for the immunohistochemistry.

Based on the observations of Suzuki et al. [27], we hypothesize that, given soluble PLTA in a plasma solution, immune complexes are formed, and herein report a novel method for detecting *P. acnes*-derived immune complexes in blood samples from sarcoidosis patients and healthy control subjects.

## 2. Materials and Methods

### 2.1. Samples

Blood plasma samples were obtained from 51 sarcoidosis patients and 35 healthy volunteers as a control between April 1995 and December 2016, at the Tokyo Medical and Dental University Hospital. Sarcoidosis was diagnosed on the basis of histologic and clinical findings following the guidelines of the American Thoracic Society/European Respiratory Society/World Association of Sarcoidosis and Other Granulomatous Disorders [28]. Table 1 shows the clinical profiles of the patients with sarcoidosis. All plasma samples from sarcoidosis patients were obtained at diagnosis without any therapeutic treatment, and no special comorbidities of patients were described in the available clinical records. All subjects provided their informed consent for participation in the study. The study was conducted in accordance with the Declaration of Helsinki, and the protocol was approved by the Ethics Committee of Tokyo Medical and Dental University Hospital (Project identification code: 877).

### 2.2. Plasma Sample Pretreatment Procedures

To detect *P. acnes*-derived immune complexes, plasma samples were pretreated by acidification, heating, and neutralization as follows. Human plasma samples (100 µL) diluted 1:4 in phosphate-buffered saline (PBS pH 7.4) were added to an equal volume of glycine buffer (100 mM glycine, 0.5 M NaCl, pH 1.0), then incubated overnight at 4 °C.

After acidification, the plasma samples were incubated for 30 s at 100 °C and then neutralized (pH 7.4) by adding 18 µL of 1 M Tris-HCl buffer (pH 13). To confirm that the glycine treatment followed by heating does not potentially remove conformational epitopes of the PLTA, each concentration of PLTA purified from *P. acnes* diluted in PBS or plasma was subjected to sandwich ELISA for detecting PLTA before and after the pretreatment. The appropriate heating duration to prevent reformation of immune complexes after neutralization was determined by changing the duration time in the same protocol.

### 2.3. Sandwich ELISA for Detecting Plasma PLTA before or after the Pretreatment

Plasma PLTA concentrations detectable before or after the pretreatment were measured by a sandwich enzyme-linked immunosorbent assay (ELISA); a mouse anti-*P. acnes* monoclonal antibody that recognizes PLTA and does not cross-react with LTA from other species [29] was used as a capture antibody for the primary reaction, and a rabbit anti-*P. acnes* antiserum raised by immunization with a whole bacterial lysate of *P. acnes* (ATCC6919) was used as a detection antibody after the primary reaction. Flat-bottomed 96-well NUNC-immuno plates (Nalge Nunc International, Rochester, NY, USA) were coated with the capture antibody (mouse ascites) diluted 1:1000 in carbonate-bicarbonate buffer (pH 9.6) at 37 °C for 60 min. Plasma samples before or after the pretreatment were added to each well and incubated at 37 °C for 60 min. After the primary reaction, the plates were incubated first with the detection antibody (rabbit serum) diluted 1:500 in PBS for 60 min at 37 °C and then with horseradish peroxidase-conjugated anti-rabbit immunoglobulins (K4003, EnVision, DAKO, Glostrup, Denmark) diluted 1:10 in PBS for 30 min at room temperature. The plates were washed with PBS containing 0.5% Tween 20 (T-PBS) before and after each step. Citrate phosphate buffer (pH 5.4) containing 0.3% o-phenylenediamine dihydrochloride (MilliporeSigma, St. Louis, MO, USA) was added to each well with 0.012% hydrogen peroxide, and then the plates were placed in a dark room and incubated for 15 min at room temperature. The reaction was stopped by adding 25 µL of 2 N HCl to each well. A Bio-Kinetics Reader (Bio-Tek Instruments Inc., Winooski, VT, USA) with a 490-nm filter was used to read the plates. The mean of 3 identical wells was used for the assay results. The plasma PLTA concentration that was detectable in each sample after the pretreatment was determined using a standard curve obtained by each concentration of PLTA purified from *P. acnes* (ATCC6919) [11] added to a control plasma sample with no detectable PLTA and detectable anti-PLTA antibodies, which was pretreated in the same manner as the samples to be examined. To examine the potential effect of the pretreatment or plasma anti-PLTA antibodies on the detection of native bacterial components, each concentration of a whole *P. acnes* lysate diluted in PBS or plasma with a high or low antibody titer was measured by sandwich ELISA before and after the pretreatment. 

### 2.4. Indirect ELISA for Detecting Plasma Anti-PLTA Antibodies

Plasma anti-PLTA antibody titers were examined for IgG, IgA, and IgM by indirect ELISA using samples without pretreatment. Flat-bottomed 96-well NUNC-immuno plates (Nalge Nunc International, Rochester, NY, USA) were coated with PLTA (0.5 µg/well) purified from *P. acnes* (ATCC6919) [8] in carbonate-bicarbonate buffer (pH 9.6) at 37 °C for 60 min. Human blood plasma samples diluted 1:200 in PBS were added to each well and incubated at 37 °C for 60 min. After the primary reaction, the plates were incubated for 30 min at room temperature with biotinylated goat anti-human IgG, IgA, or IgM antibody (AHI1309, AHI1109 or AHI1609, respectively, Invitrogen, Carlsbad, CA, USA) diluted 1:5000 in PBS and then incubated for another 30 min with horseradish peroxidase-conjugated streptavidin (P0397, DAKO, Glostrup, Denmark) diluted 1:5000 in PBS. The plates were washed with T-PBS before and after each step. Procedures for the color development and reading of the values were the same as for the sandwich ELISA described in the preceding section. Plasma anti-PLTA antibody titer is shown as the mean optimal density at 490 nm of 3 identical wells.

### 2.5. Statistical Analyses

For evaluation of differences in the plasma PLTA concentration and anti-PLTA antibody titer between the 2 groups, we used the Mann–Whitney U test. For evaluation of the sensitivity and specificity of the plasma PLTA concentration for sarcoidosis, a receiver operating characteristic (ROC) curve was plotted. All analyses were performed using GraphPAD PRISM ver. 6 (GraphPad Software, Inc., San Diego, CA, USA). *p* < 0.05 was considered statistically significant.

## 3. Results

### 3.1. PLTA Detection Recovered by the Pretreatment

**Figure 1:**
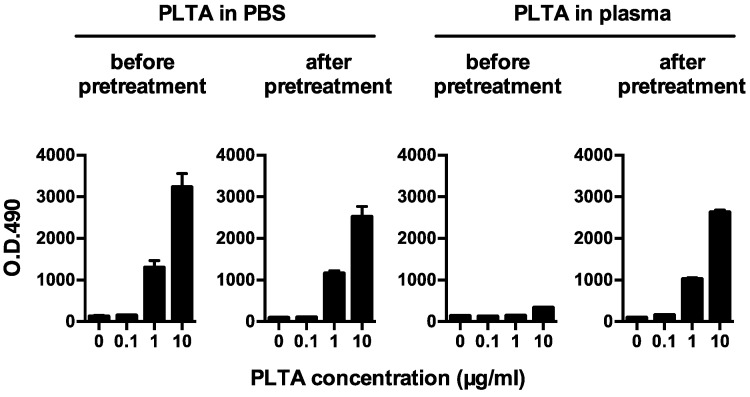
Detection of *P. acnes*-specific lipoteichoic acid (PLTA) diluted in PBS or plasma before and after the antigen retrieval pretreatment. Representative results of a plasma sample obtained from a healthy volunteer are shown. While PLTA diluted in PBS was detected in a dose-dependent manner within the range below the peak absorbance before and after the pretreatment, the dose-dependent detection of PLTA diluted in the plasma sample was inhibited before pretreatment and recovered by pretreatment. Error bars indicate standard deviation of 3 identical wells.

**Figure 2:**
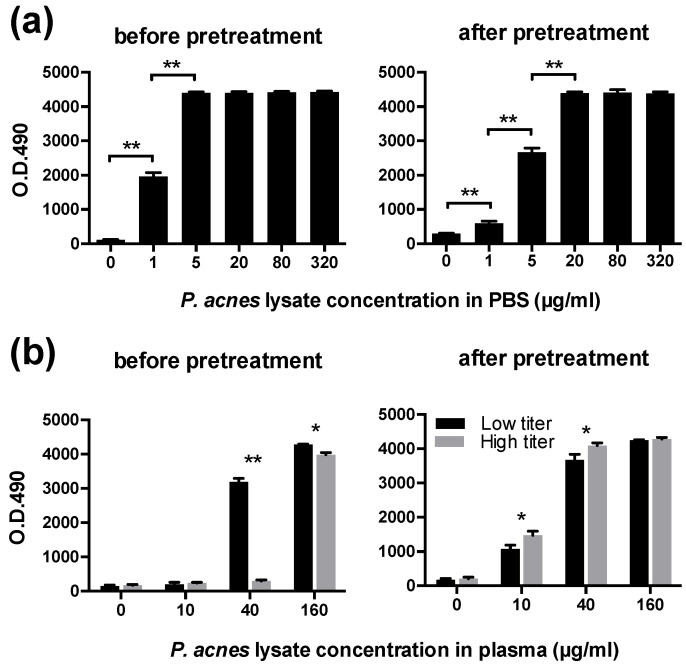
Effect of the pretreatment and plasma antibodies on the detection of native bacterial components. (**a**): A whole *P. acnes* lysate diluted in PBS was detected in a dose-dependent manner within the range below the peak absorbance before and after the pretreatment, showing slight degeneration of the bacterial component by the pretreatment. (**b**): before the pretreatment, detection of *P. acnes* lysate diluted in plasma was more inhibited in a sample with a high plasma antibody titer (gray columns) than in a sample with a low plasma antibody titer (black columns). After the pretreatment, dose-dependent detection was observed in both samples regardless of the antibody titer. Error bars indicate standard deviation of 3 identical wells. * *p* < 0.05, ** *p* < 0.001.

Before the pretreatment, PLTA diluted in PBS was detected by sandwich ELISA in a dose-dependent manner, whereas the dose-dependent detection of PLTA diluted in plasma was remarkably inhibited (Figure 1). After the pretreatment, the dose-dependent detection of PLTA in the plasma recovered and was almost the same as that of PLTA diluted in PBS. The potential pretreatment-induced degeneration of the *P. acnes* antigen was estimated to be minimal when evaluated using a whole *P. acnes* lysate diluted in PBS (Figure 2a). Plasma anti-PLTA antibody titers did not affect the assay results of the *P. acnes* antigen detectable after the pretreatment when examined using the whole *P. acnes* lysate diluted in the 2 control plasma samples with high or low antibody titer (Figure 2b).

### 3.2. Plasma PLTA Concentrations before and after the Pretreatment

**Figure 3:**
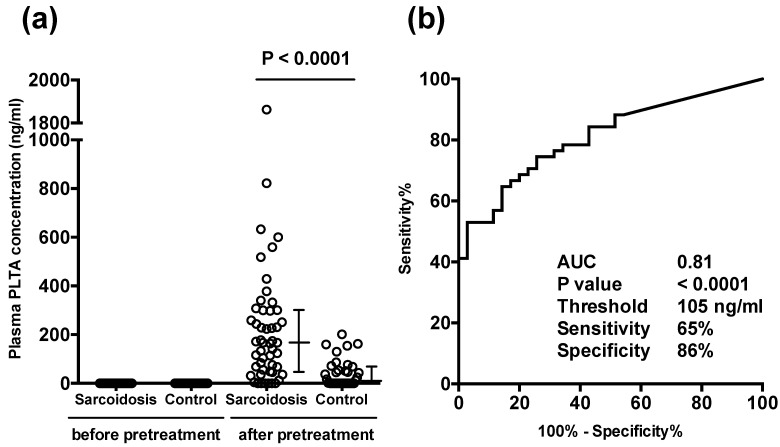
Plasma concentration of *P. acnes*-specific lipoteichoic acid (PLTA) detectable before and after the pretreatment of plasma samples from sarcoidosis patients and control subjects. (**a**): PLTA was not detected in any of plasma samples before the pretreatment. After the pretreatment, plasma PLTA was detected in varying concentrations, but at higher levels in sarcoidosis patients than in control subjects. Error bars indicate median with 25th and 75th percentiles. (**b**): ROC curve of the plasma PLTA concentration detected after pretreatment.

Before the pretreatment, PTLA was not detected in any of the samples. After the pretreatment, however, PLTA was detected in many samples indicating that the median plasma concentration of PTLA in sarcoidosis patients (*n* = 51; 167 ng/mL) was higher than that in control subjects (*n* = 35; 12 ng/mL) (Figure 3a). According to the threshold value (105 ng/mL) determined by the ROC curve (Figure 3b), a significant level of PLTA was detected in 33 (65%) sarcoidosis patients and in 5 (14%) control subjects, with 86% specificity and 65% sensitivity for sarcoidosis. PLTA was abnormally increased (>202 ng/mL) to levels greater than the maximum level of control samples in 21 (41%) sarcoidosis patients. No significant association was detected between the plasma PLTA levels and any clinical parameters of the sarcoidosis patients obtained in the study, including age and sex.

### 3.3. Plasma Anti-PLTA Antibody Titers

**Figure 4:**
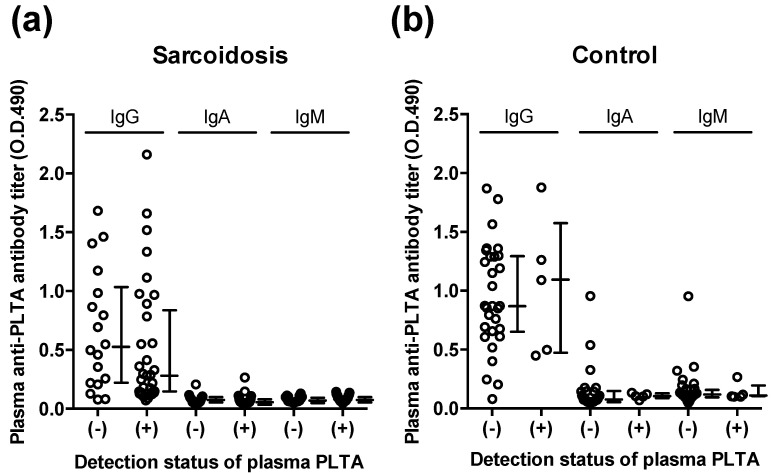
Plasma antibody titers against *P. acnes*-specific lipoteichoic acid (PLTA) in (**a**) sarcoidosis patients and (**b**) control subjects. No significant difference was found in plasma anti-PLTA antibody titers (IgG, IgA, and IgM) between samples with and without detection of PLTA. (-): negative for PLTA detected. (+): positive for PLTA detected. Error bars indicate median with 25th and 75th percentiles.

Plasma anti-PLTA antibodies were detected in all samples. In neither the sarcoidosis group nor the control group was there a significant difference in the IgG, IgA, and IgM antibody titers between samples with and without a significant level of PLTA (>105 ng/mL) detected after the pretreatment (Figure 4).

## 4. Discussion

Gram-positive bacteria including *P. acnes* contain lipoteichoic acid as a major biologically active cell surface layer component whose structure varies between bacterial species [30,31]. Lipoteichoic acid confers antigenic properties that stimulate specific immune responses in individuals infected with gram-positive bacteria.

In the present study, we established a sandwich ELISA method for measuring the concentration of PLTA using a *P. acnes*-specific mouse monoclonal antibody as a capture antibody that binds with PLTA. Human immunoglobulins (mainly IgG) also bound with PLTA coated on ELISA plates, allowing us to detect human plasma antibodies against the antigen. While anti-PLTA antibodies were detected in all plasma samples due to the commensalism of the bacterium, PLTA could not be detected in any plasma sample that did not undergo the pretreatment procedure. Although purified PLTA or a whole *P. acnes* lysate diluted in PBS was detectable in a dose-dependent manner, the dose-dependent detection of the *P. acnes* antigen diluted in plasma was remarkably inhibited; the antigen retrieval pretreatment of plasma samples developed in the present study, however, successfully recovered the dose-dependent detection. These results suggest that an epitope competition for a PLTA antigen determinant occurs between human plasma anti-PLTA antibodies raised as an infection antibody against the commensal bacterium and a mouse anti-PLTA monoclonal antibody used as a capture antibody in the sandwich ELISA for detecting PLTA.

We developed a pretreatment method for detecting plasma PLTA bound with immunoglobulins according to the observation that antigen-antibody binding can be dissociated below pH 2.3 and that the PLTA antigen determinant recognized by the capture antibody for detecting PLTA is heat resistant. By applying the pretreatment procedure to plasma samples, PLTA bound with immunoglobulins was dissociated by acidification and the anti-PLTA binding of immunoglobulins was abolished by heating the acidified samples. After neutralization, the concentration of PLTA detectable in the pretreated plasma samples could be measured by sandwich ELISA. Heating for 30 s at 100 °C before neutralization was essential to prevent the reformation of immune complexes after neutralization and did not abolish the antigenicity of the purified PLTA or whole cell lysate of *P. acnes*.

The PLTA concentration detectable after pretreatment reflects the amount of *P. acnes*-derived circulating immune complexes comprising PLTA bound with immunoglobulins in the original plasma sample. Although a low level of these immune complexes was detected in both sarcoidosis and control samples, only sarcoidosis patients (41%) had high levels of the immune complexes. In previous reports [20,21,22,23,24,25], circulating immune complexes were detected in 3% to 58% of sarcoidosis patients depending on the detection technique used. The association between *P. acnes*-derived circulating immune complexes detected in the present study and the non-specific circulating immune complexes detected in previous studies requires further study.

The conformation of the *P. acnes*-derived circulating immune complexes remains unknown. Immunohistochemical studies revealed *P. acnes* in sarcoidosis lymph nodes as small round bodies in granulomas or large ovoid Hamazaki-Wesenberg bodies in sinus macrophages suspected to be cell-wall deficient mycobacteria on the basis of their morphology [11]. *P. acnes*-derived insoluble immune complexes were detected by a unique antigen retrieval pretreatment of tissue sections in the lymphatic sinus macrophages where the PLTA and immunoglobulins are located together at the peripheral rim of the small round bodies without a cell wall structure [27]. Many *P. acnes* in sinus macrophages are bound with immunoglobulins, whereas those in sarcoidosis granulomas located in paracortical areas are predominantly free of immunoglobulins. The difference between *P. acnes* bound or not bound with immunoglobulins suggests that after intracellular proliferation of the bacterium, *P. acnes*-derived insoluble immune complexes form outside the cell and are phagocytosed by sinus macrophages; those that escape phagocytosis, however, may spread via the lymphatic system and bloodstream [19]. According to this hypothesis, *P. acnes*-derived circulating immune complexes detected in the present study may have formed in the organs in which the bacterial proliferation occurred. Because many *P. acnes* in sinus macrophages were bound with IgA and IgM [27], the circulating immune complexes in sarcoidosis patients may be *P. acnes* bound with IgG. It remains uncertain, however, whether the circulating immune complexes form against the bacteria cells themselves or the bacterial cell components after disruption of the cell.

A low level of *P. acnes*-derived circulating immune complexes was detected in some healthy control individuals. *P. acnes* is the most common commensal microorganism in the lungs and lymph nodes [32], persisting in macrophages without intracellular replication [33]. While immunohistochemical detection of *P. acnes* in granulomas is useful for differentiating sarcoidosis from other granulomatous diseases, the commensal bacterium is frequently detected outside of granulomas in both sarcoidosis and control tissues [12]. The development of sarcoidosis requires both a genetic predisposition and environmental exposure to unknown antigens [2]. A dysregulated immune response against intracellular proliferation of the commensal bacterium may cause granulomatous inflammation. In sarcoidosis patients, as well as in healthy individuals or other patients, latent *P. acnes* may be reactivated [17,18]. Further studies using plasma or serum samples from patients with diseases other than sarcoidosis are needed to elucidate the role and etiology of *P. acnes*-derived circulating immune complexes in sarcoidosis. We are now planning studies to monitor the levels of *P. acnes*-derived immune complexes in cardiac sarcoidosis patients treated or not treated with antibiotic therapy in a multicenter, open-label, randomized, controlled study named Japanese Antibacterial Drug Management of Cardiac Sarcoidosis (J-ACNES) [34].

The pretreatment method developed in the present study may be useful for detecting circulating immune complexes formed against other species-specific lipoteichoic acid structures found in gram-positive bacteria or potentially lipopolysaccharide in gram-negative bacteria, although this method cannot be used to detect immune complexes that form against protein antigens of any microorganism.

This study has some limitations. Based on the results that PLTA was not detected in all samples but was successfully detected in some samples after the pretreatment for dissociating the antigen-antibody complexes, we concluded that *P. acnes*-derived circulating immune complexes are present in some human plasma samples. PLTA bound with immunoglobulins, however, was not directly identified in the present study. Plasma, but not purified immunoglobulins raised against PLTA, was used in the analysis. Many other plasma proteins are affected by the pretreatment, however, and using only plasma does not show anti-PLTA antibodies may mask PLTA. Further studies are needed to demonstrate PLTA bound with immunoglobulins in the original plasma samples before the pretreatment.

## 5. Conclusions

The present study demonstrated for the first time that *P. acnes*-derived circulating immune complexes are present in human blood. Our findings using a newly developed pretreatment method suggest that the abnormally high levels of these immune complexes in some sarcoidosis patients may be due to local proliferation of the bacterium in the affected organs.

## Figures and Tables

**Table 1:** Clinical profiles of patients with sarcoidosis.

Clinical Characteristics	
Number	51
Subjects (male/female)	20/31
Age, years	51 ± 16
Chest X-ray Stage (0/I/II/III/IV/NA)	2/18/24/3/1/3
Angiotensin converting enzyme (U/I/37 °C) ^a^	22.8 ± 10.7
Lysozyme (µg/mL) ^b^	12.6 ± 9.4
Soluble interleukin-2 receptor (U/mL) ^c^	1235 ± 841
Pulmonary function test	
% Forced vital capacity (%) ^d^	105 ± 14
Forced expiratory volume in 1 s (%) ^e^	80 ± 10
Bronchoalveolar lavage analysis	
total cell (10^5^ cell/mL)	2.7 ± 1.6
macrophage (%)	66 ± 20
lymphocytes (%)	32 ± 20
CD4/CD8 ratio ^f^	5.9 ± 3.9

Normal range: ^a^ 8.3–21.4, ^b^ 5.0–10.2, ^c^ 220–530, ^d^ > 80, ^e^ > 70 and ^f^ < 3.5.

## Data Availability

Not applicable.
